# Factors influencing the use of clinical guidelines by general practitioners working in a setting of complex multimorbidity: a case study by interviews

**DOI:** 10.1186/s12875-018-0834-2

**Published:** 2018-09-11

**Authors:** Jeremy A. Jones, Carole A. Reeve

**Affiliations:** 1 Northern Territory General Practice Education (NTGPE), National Remote Health Precinct, 5 Skinner Street, Alice Springs, Northern Territory, 0870 Australia; 2 James Cook University, 1 James Cook Drive, Townsville City, Queensland, 4811 Australia

**Keywords:** Chronic disease, Guidelines, Aboriginal, Continuity of care, Electronic health records, Implementation

## Abstract

**Background:**

The Central Australian Remote Practitioners Association Standard Treatment Manual (CARPA) contains protocols for primary health care in remote Central Australia. This context stands in stark contrast to the mainstream settings in Australia and features an Aboriginal population with very poor health status, powerful social determinants of health, geographical isolation and high turnover of health practitioners. The manual consolidates the core elements of national guidelines, particularly as they pertain to Aboriginal health care, into a single document. The aim of this study is to explore factors that promote or impede the use of CARPA by general practitioners (GPs) in Central Australia, with a particular focus on chronic disease management.

**Methods:**

In-depth interviews were conducted with GPs and GP registrars employed in the provision of Aboriginal health care in Central Australia. Interview transcripts were thematically analysed from a critical theory perspective.

**Results:**

11 GPs and 3 GP registrars from the two major Aboriginal primary health services in Central Australia were interviewed.

The dominant theme in the data was that poor continuity of care impeded the use of CARPA. The second-most dominant theme was that electronic health record systems enhanced the use of CARPA in some ways, and impeded its use in others. Other factors influencing the use of CARPA included the culture of the health service organisation, GPs’ first impressions of CARPA, the accessibility and usability of CARPA, and GPs’ confidence practicing in such a unique environment.

**Conclusions:**

This study identifies factors from multiple domains that influence the use of best practice guidelines in the delivery of chronic disease care. It demonstrates that such factors may not be purely ’enablers’ or ’barriers’, but may be a mixture of both. It highlights the critical role of continuity of care and the potential benefits and pitfalls of using electronic health records in providing chronic disease care. This study provides empirical insights that can be used to improve chronic disease care.

## Background

### The gap between evidence and practice

“*Evidence based medicine is the conscientious, explicit, and judicious use of current best evidence in making decisions about the care of individual patients. The practice of evidence based medicine means integrating individual clinical expertise with the best available external clinical evidence from systematic research.*” [1]

Clinical practice guidelines have become a primary means of disseminating best evidence to clinicians. Clinical practice guidelines are “systematically developed statements to assist practitioner and patient decisions about appropriate health care for specific clinical circumstances” [2]. When used, clinical practice guidelines improve patient outcomes and optimise use of limited health-care resources [3].

Unfortunately, many patients do not receive medical care informed by the best available evidence. This is a well-recognised problem. In Australia [4] and elsewhere [5], guidelines are followed in just over 50% of the general practice encounters in which they are applicable.

Implementation science seeks to understand why the gap between evidence and practice exists and how to minimise it [6, 7]. It recognises the gap is multi-factorial in origin, arising not only from factors unique to each practitioner, but also from many external factors. These factors are often context-specific. Identifying and understanding these factors is crucial for moving practice towards the ideal [8].

### The Central Australian context

The remote region of Central Australia is extremely large, covering more than 10% of Australia’s land mass. It has a small population of approximately 50,000 people, 45% of whom are Aboriginal. A majority of the Aboriginal population live in communities that are very geographically isolated [9–11]. Despite the geographical isolation, the Aboriginal population is highly mobile within the region [12].

This geographically-isolated Aboriginal population also experiences cultural and language isolation. Many people maintain strong connections to their traditional cultures, which inform their concept of health as a broad sense of holistic well-being and inform their beliefs about illness causation and cure [13, 14]. Aboriginal languages remain widely spoken and are usually the first languages spoken by children [15], who often go on to have poor English literacy [16]. Interestingly, there is no corresponding word for ‘health’, as understood in western society, in Aboriginal languages [17].

The health status of the Aboriginal population of Central Australia is very poor compared to the rest of Australia. Social determinants of health are a significant contributor to this health status [17]. Diseases which are normally associated with poverty, such as scabies and rheumatic heart disease, occur at high rates [18, 19]. Rates of chronic disease such as diabetes, hypertension and renal disease are several times the Australian average [10, 20]. For example, 16.2% of Aboriginal people in Central Australia have diabetes, compared with a national Australian prevalence of 5.4% [21]. Amongst those older than 50 years, the rate of multimorbidity exceeds 60% [20].

Aboriginal primary health care services in Central Australia have characteristic features. They often operate on a walk-in basis, rather than by appointments, and patients will typically see any available health practitioner. The majority of direct primary health care in remote communities is provided by remote area nurses (RANs) and Aboriginal Health Practitioners (AHPs), with general practitioners (GPs) often acting in a supportive role on site or remotely. There is a consistently high turnover of health practitioners, especially in the most remote locations [22, 23]. All services use electronic health record systems, and publish data to a central electronic repository [24].

In 1992, the Central Australian Rural Practitioners Association first published “a collection of protocols for the management of common conditions seen in remote (mainly Aboriginal) health practice”. The aim was, and continues to be, to promote standardised, high quality and evidence-based care in the region [25, 26]. It was called The Standard Treatment Manual, but became known simply as CARPA (and referred to herein as such). Primary health care practitioners have been central to the development, evaluation and updating of the manual [26, 27]. After several revisions, the manual now covers a broad range of topics, including child health, emergency medicine, chronic disease and sexual health. The content is based on the best available evidence or, where evidence is lacking, expert opinion [28, 29]. The content is consistent with existing Australian clinical practice guidelines [27] and draws upon their Aboriginal and Torres Strait Islander specific content. The editors of CARPA aim to provide a “simple, easily portable manual” “without compromise in the content” for use by AHPs, RANs, GPs and allied health professionals [26].

CARPA has a prominent role in Aboriginal primary health care in Central Australia. All government and non-government Aboriginal primary health care services consider CARPA to be an important instrument for achieving standardised and evidence-based care and so have policies directing health practitioners to adhere to CARPA [30]. Furthermore, legislative provisions in the Northern Territory grant RANs and AHPs working in remote locations the right to prescribe medication consistent with CARPA recommendations [31]. In the absence of these provisions, only GPs would have prescribing rights. A high proportion of health practitioners use CARPA on a regular basis [30].

### Objective

This study sought to identify key factors that promote or impede the use of CARPA by GPs working in Aboriginal health in Central Australia. It is the authors’ opinion that the chronic disease recommendations of CARPA are the most difficult to apply, and therefore these recommendations were a particular focus of the study.

Similar studies have been conducted for other guidelines, but in mainstream clinical contexts [32–34]. Several large assessments of CARPA have been undertaken [30, 35]. However, those assessments focused on a narrower range of factors than this study.

## Methods

This case study was conducted with a realist paradigm [36], expecting that the synthesis of the perspectives of participants would provide deeper insight.

The author-researchers are both GPs who have previously used CARPA in Aboriginal primary health care. JJ was the principle researcher and sole interviewer. He undertook the majority of his recently-completed GP training in Central Australia, undertaking training terms at both of the health services from which participants were recruited (see below). JJ knew some of the GPs invited to participate and therefore maintained a formal decorum throughout the research process. CR has worked extensively in remote health across northern Australia both as a GP and as a public health physician with an interest in strengthening health systems to improve health outcomes.

Ethics approval for this research was granted by the Central Australian Human Research Ethics Committee.

### Recruitment

Research participants were sought from the two major providers of Aboriginal primary health care in Central Australia: the Northern Territory Government’s Primary Health Care and the Aboriginal community-controlled Central Australian Aboriginal Congress (referred to herein simply as Congress). Primary Health Care provides health services to the majority of remote locations outside Alice Springs and employed 15 GPs and 2 GP trainees at the time of study. It gave GPs permission to participate in the research during regular work hours. Congress is the major provider of Aboriginal primary health care in Alice Springs and employed 14 GPs and 8 GP trainees at the time of study. Congress is an active contributor to the development of CARPA.

All GPs who had worked in the participating organisations for more than 6 months were eligible to participate. Convenience sampling was used for logistical reasons. Recruitment presentations were made by JJ to a meeting of GPs at both each organisation in April 2016, followed up by a recruitment email distributed by each organisation’s GP manager. Recruitment efforts were restricted to this, as the researchers did not want GPs to feel coerced to participate based on existing relationships.

A sample size requirement of 15 was predicted based on similar previous research [37]. The final sample size was considered adequate when saturation was reached, as evidenced by no new themes emerging.

Participants gave formal written consent to participate in the study and, where quoted, were given the opportunity to review their quotations in context before publication.

### Data collection

Between May and July 2016, an in-depth semi-structured interview was conducted with each participant. To enhance interview consistency, a simple interview guide of four questions was used:

1. What do you understand to be the role of CARPA?
2. How do you use CARPA in your day-to-day work?
3. How do you use the chronic disease chapter of CARPA?
4. [Optional] If you had a context-specific document of a similar nature as CARPA in an urban general practice, would it be easier or harder to use? Why?

Data were recorded in hand-written notes and by audio recordings. Recordings were transcribed by a transcription service with whom JJ had entered a confidentiality agreement. The written notes were scanned to an electronic file before being shredded. The scanned notes, together with the interview audio and transcripts, were stored on JJ’s password protected laptop.

### Data analysis

Each interview was considered a unit of study. Data analysis began during the interview, with the interviewer proposing a summary of the main themes towards the end of each interview and providing the interviewee with the opportunity to correct or clarify the summary. The section of each transcript containing that summary, together with the afore-mentioned scanned notes, were quarantined until all interviews were analysed, and these two data sources were used to check for analysis integrity.

Both researchers independently reviewed the transcripts of the first four interviews using an open-coding approach. Afterwards, the codes that each researcher had used were compared and initial themes discussed. The discrepancies between the researchers’ use of codes were minimal and were resolved through discussion.

**Fig. 1:**
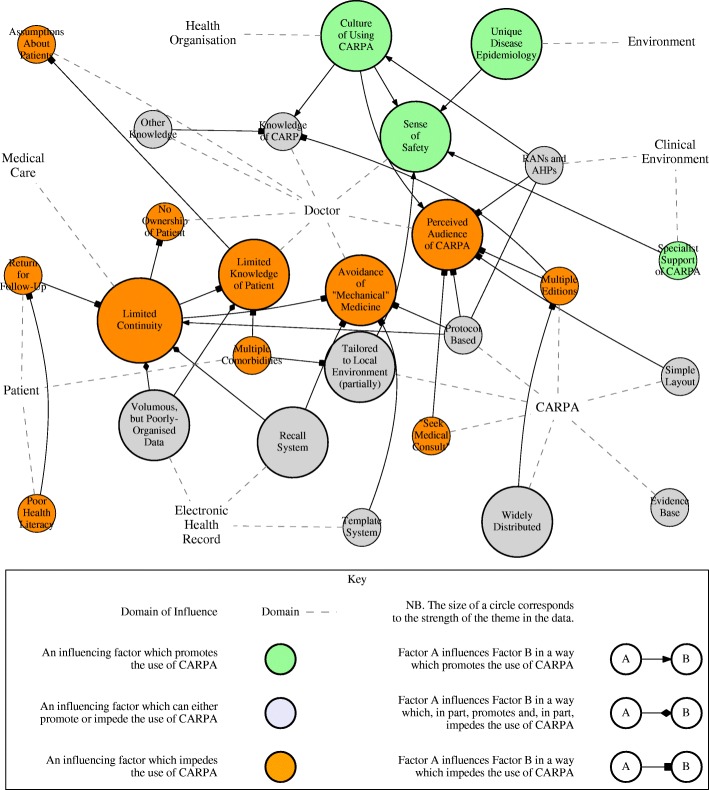
Simplified graph of factors influencing the use of CARPA and their interactions

All interviews were coded by JJ using the qualitative data analysis tool RQDA [38]. A count of new codes arising in each interview helped identify saturation. A Graphviz graph [39] of individual codes and their interactions helped the authors to visualise central factors and how they coalesced into major themes. A simplified version of this graph, showing major and minor themes, is shown in Fig. 1. A matrix of the frequency of individual code use per interview also helped the authors visualise the major themes. JJ and CR discussed new themes as they emerged.

## Results

Fourteen GPs and GP registrars participated in the research. Nine participants were employees of Primary Health Care (eight GPs and one registrar) and five were employees of Congress (three GPs and two registrars). For the GPs, the average length of career in general practice was twenty one years and the average length of time using CARPA was ten years. The three GP registrars had been working with CARPA for seven months, on average. The primary researcher knew four of the participants well and four casually; six participants were previously unknown to the primary researcher.

The average length of interview was 55 min.

Subjectively, saturation of themes was approached by interview six and reached by interview ten. This assessment was supported by the count of new codes introduced by successive interviews (see Table 1). The major themes raised by participants from Primary Health Care and those from Congress were identical. The few discrepancies in how these themes were expressed are highlighted below.

**Table 1:** Introduction of new codes by interview

Interview	1	2	3	4	5	6	7	8	9	10	11	12	13	14
New codes	69	20	13	20	19	11	23	13	7	4	5	1	1	4

The illustrative quotations included in the rest of this section are from the interview transcripts.

### Continuity of care

*“Their care is not a continued care.”* (Participant 1)

The dominant theme in the data was the need for continuity of chronic disease care to enable the most effective application of the guidelines. In this paper, ‘continuity of care’ refers to the smooth and coordinated flow of health care between different health care settings and health care providers, rather than to patients seeing the same practitioner over a period of time [40]. This is consistent with how participants used the phrase: all participants spoke about ‘continuity of care’ in the first sense; only a few spoke about it in the second sense. Without continuity of care and, in particular, the health records and patient follow-up needed to achieve it, participants felt unable to make the best use of CARPA.

*“I’d say, by and large, most of the patients, I don’t think I would have met before. Even coming to the end of six months, I’d say, most of the time, they’re new patients.”* (Participant 2)

*“Perhaps even the majority of health encounters are anonymous. In other words, the provider doesn’t know the patient particularly well.”* (Participant 3)

Whilst this theme was raised by all participants, it was noticeably more prominent in the data from Congress GPs than from Primary Health Care GPs. The reason for this discrepancy was not apparent in the data.

The data highlighted three important elements of continuity of care, as discussed below: the GPs’ knowledge of the patient, the provision of follow-up care, and the health literacy of patients.

#### Knowing the patient

All participants spoke about the importance of knowing the patient’s clinical journey – what had happened in the past and what might be expected in the future. Many participants explicitly stated that, in order for their care to be effective, they needed to tailor the recommendations of CARPA to each patient’s unique circumstances. All participants spoke about their heavy reliance on the electronic health record for knowledge of the patient. However, they reported that although the electronic health record stored necessary patient data, the data was poorly organised, making the task of piecing together the patient’s story an onerous one.

*“I’ll try and look in [the electronic health record], but obviously if there’s so much there it’s hard to see what really is important.”* (Participant 5)

*“If I had the time and took the time, I would usually take about an hour [to piece together the story] for people who had chronic health conditions.”* (Participant 6)

#### Follow-up

All participants spoke about the importance of follow-up in providing chronic disease care, as also emphasised by CARPA. However, participants frequently spoke about the frustration of patients not returning for follow-up. If participants tried to explain this patient behaviour, they spoke of poor health literacy and chronic disease care not being on the patient’s agenda.

*“It’s hard to know the value of trying to intervene for certain things if you’re not confident of follow up, I suppose.”* (Participant 2)

*“You offer, but you never get them back or hardly ever. Not in the time-frame that [you want].”* (Participant 7)

*“And then to act on what you find … [t]hat’s a second step where they have to come back. It never happens.”* (Participant 8)

*“Because they don’t come back. I would say, the main issue is getting clients back in for non-acute problems.”* (Participant 8)

#### Health literacy

Most participants stated that low health literacy amongst patients influenced patient behaviour, especially returning for follow-up.

*“There seems to be a significant lack of understanding [by patients] of the implications of [chronic] disease.”* (Participant 7)

*“There’s a big problem in that a lot of our patients, their health literacy levels would be extremely low so it is quite challenging to really try and explain what’s going on. … Most people think if they feel fine they’re okay, so they’re more likely to come in when they’re feeling unwell.”* (Participant 5)

*“The main issue is getting clients back in for non-acute problems … is that they don’t know what’s going on.”* (Participant 8)

### Other key factors

Six other major themes emerged from the data.

#### Electronic health record systems

The second-most dominant theme in the data was the role of electronic health record systems and how they both promoted and impeded the use of CARPA. The role of the systems as data repositories has been mentioned above. The role of data entry templates and automatically-generated recalls was raised by all participants. Templates, designed to mirror CARPA protocols, were seen as too prescriptive and not flexible enough to be tailored to particular clinical encounters. Several Congress GPs stated that they were frustrated by discrepancies between data entry templates and CARPA’s recommendations. Recalls were seen as an important tool in providing continuity of care, but were seen as being too numerous and poorly coordinated. Most participants reported that, practically, templates and recalls obviated the need for them to refer to CARPA directly.

*“It’s easy to almost to lose sight of the wood for the trees, there’s so many of these electronic prompts.”* (Participant 9)

*“[The recall system] alerts you as to what the requirements are for that’s person’s condition.”* (Participant 7)

*“I’m using the recalls for chronic disease rather than the actual going back to CARPA.”* (Participant 10)

*“To be honest I don’t know [what’s in the chronic disease chapter of CARPA]. Because I’m doing so many chronic disease reviews I just follow what’s on the screen.”* (Participant 11)

*“Most of the time [the electronic health record system] tells me what to do, so I don’t need to have a look at the CARPA for confirmation”* (Participant 12)

Primary Health Care and Congress use different Electronic Health Record Systems. The user experience of Primary Health Care’s system is heavily dependent on the speed of the Internet connection available at the clinical site. Most Primary Health Care GPs reported that, when working remotely, a sluggish computer interface detracted from their capacity to provide health care, particularly when consulting with a patient.

#### Tailoring guidelines to the individual patient

Nearly all participants spoke about the need to tailor the advice in guidelines to the individual patient. Many spoke about how they felt the protocol-oriented nature of CARPA was too restrictive. This issue was often raised in conjunction with the nature of electronic health record templates, as discussed above. A few participants felt that CARPA addresses individual chronic diseases in silos and does not give appropriate consideration to the complexity of caring for a population with multimorbidity.

*“[G]eneric plans don’t work, you actually have to highly individualise them.”* (Participant 4)

*“There are often no clear-cut answers for their problems. Even if you have the guidelines, about lipids, for example, it’s not that simple. It’s not just, “I apply my guidelines on you”. No, [you have to consider] the specific person.”* (Participant 8)

*“A lot of situations are more complex than CARPA can handle because of multiple comorbidities.”* (Participant 5)

*“[Templates offer] a fairly generic plan, if you like, for each type of problem that they have.”* (Participant 3)

#### Organisational culture

Participants universally reported strong organisational cultures promoting the use of CARPA. Contributors to this culture included: directives to use CARPA in employment conditions and during workplace orientation; the use of CARPA by colleagues, particularly AHPs and RANs; modelling of use of CARPA by senior staff and registrar supervisors; and frequent reference to CARPA at clinical meetings. Furthermore, support of CARPA content by local hospital specialists reinforced the organisational culture.

*“There’s a lot of emphasis of CARPA from all sorts of people.”* (Participant 9)

*“Because people refer to [CARPA] on a day-to-day basis from all different areas, it provides me with a strong sense of adopting that as the primary standard.”* (Participant 7)

*“It’s actually written into the position description for the doctors, so if you’re not following it, then you’re not actually doing your job.”* (Participant 13)

Interestingly, four of the five Congress GPs reported they had taken the time to familiarise themselves with CARPA before beginning work. These disclosures were unprompted. In comparison, only one of the nine Primary Health Care GPs reported doing likewise. The reason for this discrepancy was not apparent in the data.

#### Clinical confidence

Many participants reported that CARPA increased their confidence to practice in certain circumstances, such as when they needed to respond to situations or diseases that they were unfamiliar with, when they needed to delegate care to RANs or AHPs or when they were worried about their practice being scrutinised by others. In these sorts of situations, a desire for clinical confidence promoted the use of CARPA.

*“There’s certain conditions such as rheumatic heart disease and that which we find here which we wouldn’t see very often in other places.”* (Participant 5)

*“One area that I felt really out of my depth was just diagnosing rheumatic fever, and it was just there in the book.”* (Participant 13)

*“I can use CARPA to protect myself, if somebody asks me why I [provided treatment in] this way.”* (Participant 1)

*“Because people won’t criticise you. …“Well, look, I was just following the guidelines of what was agreed for the Territory.””* (Participant 13)

*“We could always go back to CARPA and say, “Look, this is how we’re doing it and that’s what’s in the book. So leave us alone.””* (Participant 6)

Conversely, participants reported that when they were confident in their existing knowledge and skills, they were unlikely to refer to CARPA. This was reported particularly by participants with extensive professional experience prior to beginning work in Aboriginal health in Central Australia, but it was also reported by GP registrars.

*“I don’t think I need to use CARPA in that regard, because I already have the knowledge for that kind of thing.”* (Participant 12)

*“CARPA’s there more or less as a backup; if I’m really not sure about how to treat them I’ll look at CARPA.”* (Participant 4)

*“I use guidelines for management of head injuries, management of pneumonia that I would know from my other work.”* (Participant 5)

#### Accessibility and usability

Participants reported that hard copies of CARPA were readily available in most clinical contexts. All but two participants preferred using a hard-copy edition, saying that it was easy to navigate and did not compete with computer screen real estate and that the Internet edition was unusable in locations with limited Internet access. Participants consistently expressed appreciation for having a single ‘go to’ resource with straight-forward and practical content. All of these factors promoted the use of CARPA.

*“I don’t think I’ve ever not been able to find a CARPA. They’re usually in the houses as well. It’s a bit like the Gideon’s Bible in a hotel.”* (Participant 7)

*“It just doesn’t take me as long to find it in CARPA.”* (Participant 13)

*“To get so much information into something that is still a usable size is excellent.”* (Participant 10)

*“The words used are very simple, easy to understand … it’s not very complicated, it’s easy to follow as well.”* (Participant 12)

*“[CARPA is] an easy place to find something without having to look up too many other things.”* (Participant 13)

*“That’s probably one of its advantages is that it’s quite practical for a lot of things.”* (Participant 2)

#### First impressions

Many participants reported that their first impressions of CARPA were that it was not a resource for GPs and that this impeded their use of CARPA. Reasons given for this included the simple layout and straight-forward nature of the protocols, as well as the safety clause intended for AHPs and RANs — “Refer to doctor” — at the end of most protocols. Most of these participants reported that, with time, they came to recognise the value of CARPA for their own work.

*“I think probably when I first started using it, I didn’t really quite perceive how useful it was going to be. Because I guess if you think about the target audience for CARPA, it is written probably for a non-medical audience or at least, that’s, you know, when you first look through it, that’s the impression you get. Because you know, there’s lots of points at which – when it comes to the, sort of, finer aspects of management, there’s the kind of advice that you should refer to doctor. So it, kind of, gives the impression that the book is essentially aimed at health centre staff. But over time, you come to realise that actually it is the number one resource for the doctor as well.”* (Participant 9)

## Discussion

The objective of this study was to identify key factors that promote or impede the use of CARPA by GPs working in Aboriginal health in Central Australia. Organisational culture, and accessibility and usability were promoting factors. The difficulty in achieving continuity of care and first impressions of CARPA were impeding factors. Interestingly, the electronic health record systems and clinical confidence were factors which, in part, promoted and, in part, impeding the use of CARPA.

The word ‘use’ in the research question was ambiguous: participants interpreted it as both the simple act of referring to CARPA and the skillful tailoring of its recommendations to an individual patient’s situation. Several participants explicitly emphasised the need to skillfully tailor recommendations within guidelines to individual patients, a sentiment also expressed in guideline literature (for example, [2, 3]). As stated above, participants described how the nature of CARPA’s recommendations, which often take the form of step-wise protocols and which mightn’t consider the complexity of a patient’s needs, together with inflexible electronic data entry templates, impeded this skillful ‘use’ of CARPA.

Many, but not all, of the themes identified in this study have been raised elsewhere [32–34, 37] and fall within domains commonly included in theoretical frameworks of implementation research, such as the Consolidated Framework For Implementation Research (CFIR) [41]. Using the CFIR as a reference, for example, themes relating to the practitioner (existing knowledge, and clinical needs), the intervention (first impressions, and accessibility and usability of CARPA) and the organisation (its culture and supporting resources such as electronic health record systems) could have been anticipated.

A strength of this study was that it identified two novel themes arising from the inter-relationship of multiple domains of influence: continuity of care and clinical confidence. Continuity of care runs across the domains of patient, practitioner, intervention and organisation. Clinical confidence runs across the domains of practitioner, environment and organisation [41]. Practitioner ‘need’ has been discussed elsewhere (e.g. [8, 41]), but only as a factor of the practitioner and in a way which does not capture the richness of the theme of ‘confidence’ raised here. It seems to the authors that implementation research literature tends to discuss domains of influence in isolation, and not factors that arise at their intersection.

Another strength of this study was that it had a more open design compared to previous assessments of CARPA which used predefined conceptual frameworks. The most recent of these other assessments, published during the data collection phase of this study, also identified the unexpected theme of clinical confidence, but did not identify the major theme identified here, namely continuity of care [35]. Similarly, it did not identify the influence of electronic health records on the use of CARPA. It is likely that this study’s open design led to the identification of factors which may not, at first glance, seem to relate to the use of CARPA.

The theme of continuity of care was unexpected by the authors because it’s impact on the implementation of chronic disease guidelines has not, to the authors knowledge, been previously highlighted in the implementation science literature. Certainly, the role of continuity of care in relationship to chronic disease care is prominent in medical literature [42], but it has not been explicitly considered as a fundamental underlying factor when implementing chronic disease guidelines. Continuity of care may have been an unstated assumption in previous implementation science research which has been unearthed in the unique context of this study. This study shows how continuity of care is fundamental to the successful implementation of chronic disease guidelines.

It is interesting that, whilst all participants spoke about the role of Electronic Health Record Systems in capturing a patient’s clinical journey, none spoke about the potential role of patients in providing the same information. The reasons for this can only be speculated upon, but may relate to perceptions of patient agency, patient health literacy, or cultural or language barriers. In fact, conspicuous by its relative absence was discussion of cross-cultural and language/communication impediments, which the authors had expected would be more prominent.

Active participation of patients in health care is a central tenent of chronic disease care models and is the focus of patient-centred care [43, 44]. The data in this study suggests that, in general, Aboriginal patients in Central Australia are not active participants in the care of their chronic disease. However, there is evidence from elsewhere in Australia that patient-centred care improves health outcomes in Aboriginal populations [45]. How health services in Central Australia might provide more patient-centred care, and how CARPA as a guideline might support this, are questions warranting further consideration.

The data from this research suggest that giving greater recognition to traditional Aboriginal knowledge is one way of improving patient-centred care. Participants described their patients’ knowledge of health and illness in terms of deficit only, consistent with a ‘deficit model’ of health literacy [46]. This perspective risks underestimating the value of traditional knowledge and its role in informing the attitudes of many Aboriginal patients towards health and illness. The data does not suggest why GPs hold this ‘deficit’ perspective.

The authors of this study had expected the theme of multimorbidity to be dominant. Instead, it was raised by only a few participants. Commentators elsewhere have discussed the challenge of integrating the advice from different guidelines when caring for patients with multimorbidity [47].

The study had a small sample size with the inherent risk of participant bias. However, except for the afore-mentioned discrepancies, the results of this study correlate very closely with existing literature [32, 33]. The uniqueness of the study context may reduce the applicability of these findings to other contexts.

## Conclusions

This study demonstrates that multiple factors influence how health care pracitioners use best-practice chronic-disease guidelines. In seeking to promote best practice, such influencing factors must be recognised and addressed by policy makers, health service organisations and the authors of guidelines.

This study identifies several important factors that influence chronic disease health care. Most prominent among them is the critical role of continuity of care in chronic disease care. This study emphasises that electronic health record systems influence continuity of care by how they capture and represent each patient’s clinical journey. Furthermore, this study highlights that continuity of care is impeded when patient follow-up fails and when clinician and patient don’t have a shared knowledge of health and illness.

Finally, this study has demonstrated that factors influencing implementation might not be purely ‘enablers’ or ‘barriers’, but may be a mixture of both.

These empirical findings provide a deeper understanding of the key factors influencing the adherence to chronic disease guidelines by primary care doctors. They provide insights that may help to close the gap between knowledge and practice and so improve health outcomes for patients with complex chronic disease.

## Abbreviations

AHP: Aboriginal health practitioner
CARPA: The central Australian remote practitioners association standard treatment manual
GP: General practitioner
RAN: Remote area nurse
